# Heterogeneity across Australian ICT policies for education of gifted students

**DOI:** 10.1016/j.heliyon.2023.e19103

**Published:** 2023-08-11

**Authors:** Rabee Alqahtani, Mohammed Ayid Alqahtani

**Affiliations:** a School of Education, University of Wollongong, 2500, Australia; b Educational Technology Department, College of Education, University of Bisha, Bisha 61922, Saudi Arabia

**Keywords:** Gifted student education, Australian ICT policies, Document analysis

## Abstract

This paper critically evaluates the current status of Information and Communication Technology (ICT) policies for gifted education in Australia, highlighting the lack of a comprehensive understanding and analysis of the heterogeneity across these policies. Despite the increasing recognition of the importance of ICT's importance in meeting the unique needs of gifted learners, there is a dearth of research systematically investigating the variations in policy frameworks, approaches to ICT integration, and their impact on educational outcomes for gifted students. This research problem necessitates an in-depth exploration of the heterogeneous nature of Australian ICT policies and their implications for promoting equitable access and fostering effective educational experiences. To address this gap, relevant policy documents and research articles on Australian ICT policies for gifted education were collected via Scopes, Web of Sciences, and Google Scholar. The search yielded six policy documents and 14 critical research articles. Our findings reveal that while Australia has definite policies for educating gifted students, these policy documents rarely mention ICT policies specifically related to gifted education. In fact, many elements of these policies only imply use of ICT as facilitation factors. Research articles highlight the inadequacies of sufficient ICT policies for gifted children in rural and remote locations, especially as they pertain to Aboriginal peoples, thereby violating the principles of equity in education. Moreover, the performance of gifted students on Science, Technology, Engineering, and Mathematics (STEM) subjects has been declining on international tests, indicating the inadequacies of Australian policies for the education of gifted students in these areas. Here, ICT policies can provide substantial solutions to address these challenges.

## 1 Introduction

Providing effective education for gifted students is a significant challenge faced by Australia and many other countries. In any population, only a small percentage of individuals stand out as gifted, possessing exceptional abilities that require differentiated educational programmes and services to meet their unique learning needs [1]. Estimates suggest that 2–5% of the population can be classified as gifted, with Australia reporting a total of 720,150 gifted students in September 2019, including all sectors and significant foreign enrolment [2].

Francoys Gagné’s [3] Differentiated Model of Giftedness and Talent (2.0) provides a comprehensive definition of giftedness. According to this model, giftedness “encompasses a range of abilities” and “recognises that giftedness is only potential and must go through a transformative process in order to become a talent” (ACARA, 2013). However, the process of identifying gifted students in Australia is far from uniform [4,5], with variations across states, districts, and even individual schools. This absence of a nationally applied standard poses a significant challenge in ensuring proper identification and provision of education that meets the needs of gifted students, which can include children and adolescents from diverse backgrounds, such as different ethnicities, races, genders, cultures, and socio-economic statuses.

Two key steps in meeting this challenge involve proper identification of gifted students and investing in the delivery of gifted education itself. In addition, leveraging special tools and teaching methods, including educational technologies, can significantly contribute to the realisation of gifted students' full potential. Information, Communication, and Technology (ICT) offer unprecedented opportunities to enhance the educational experiences of gifted students. By fostering high-level thinking skills such as creative, critical, and reflective thinking [6], facilitating connections with experts worldwide, and promoting collaboration among peers, ICT can significantly contribute to the development of gifted students. Further, ICT provides teachers with online communities where they can share resources and innovative practices in gifted education, such as The National Association for Able Children in Education (NACE) [7], the Support Society for Children of Higher Intelligence (CHI), and the National Association for Gifted Children (NAGC). ICT's transformative potential in gifted education aligns with the broader impact it has had in various fields, urging educational institutions, administrators, and teachers to reconsider their roles and visions for the future [8]. Additionally, ICT has shown promise in improving access, relevance, and quality of education, particularly in developing countries where they can address challenges of isolation and expand access to knowledge [9].

However, determining best practices in gifted education is a complex task, primarily due to the limited research evaluating the effectiveness of existing provisions [10]. The diverse nature of gifted and talented students, considering their varying degrees and types of exceptional abilities, further adds to this complexity [11]. Moreover, the adoption of standardised practices is complicated by the diverse approaches employed across educational contexts, including different placement strategies for gifted students. Additionally, variations in teacher backgrounds and training significantly impact the delivery of effective education to gifted students [12]. Gifted education is not a homogenous affair, and the students and teachers cannot be characterised in a uniform manner. In light of these challenges, exploring the potential benefits of ICT for gifted students requires a nuanced understanding of the complex dynamics at play.

To effectively utilise ICT in gifted education, it is important to consider specific educational strategies that go beyond technological availability. Stratford and Brown [13] emphasise the need to shift the focus from a technocentric perspective that solely emphasises the technology itself, to one that emphasises the social practices associated with ICT. Merely incorporating technology into the classroom without considering its pedagogical value is insufficient for improving educational outcomes. Therefore, articulating educational strategies that empowers teachers to make effective use of ICT in supporting the teaching and learning objectives of gifted students is crucial. The Ministry of Education's strategy document entitled Digital Horizons: Learning Through ICT (2002–2004) outlines four key areas of focus: (1) students - enhancing learning experiences and outcomes for students through ICT; (2) providing support for educators on how to integrate ICT into the curriculum and management practices; (3) improving the efficiency and effectiveness of ICT in management and administration; and (4) fostering partnerships with communities, businesses, and other stakeholders.

ICT is a global phenomenon that encompasses different cultural and social perspectives [14]. In Australia, the ubiquity of ICT devices and Internet connectivity in schools has been prevalent for over two decades now (OECD, 2005). However, it is important to note that even basic notions and key terms in ICT curricula can vary across states and territories in Australia. This diversity reflects the multifaceted nature of ICT implementation and highlights the need for a comprehensive understanding of the various approaches and perspectives in order to effectively harness its potential for gifted education.

By considering specific educational strategies and recognising the diverse cultural and social perspectives surrounding ICT, educators and policy makers can navigate the complexities associated with integrating ICT into gifted education. This approach ensures that the use of technology aligns with pedagogical goals and addresses the unique needs of gifted students, ultimately leading to enhanced educational experiences and outcomes.

## 2 The aim of the paper

This study aims to address the aforementioned challenges by investigating the heterogeneous nature of ICT policies for gifted education in Australia. By analysing relevant policy documents and research articles, the study aims to identify gaps, variations, and shortcoming existing policies, particularly concerning the integration of ICT. The research will specifically focus on promoting equitable access to gifted education and enhancing educational outcomes, with a particular emphasis on Science, Technology, Engineering, and Mathematics (STEM) subjects. Six policy documents were collected from publicly available state websites, and 14 research articles critically evaluating Australian ICT policies were collected by searching Scopes, Web of Sciences and Google Scholar.

## 3 Research questions

This research article analyses ICT policies related to gifted education in public documents from Australian National and State governments and surveys previous literature evaluating these policies. Thus, the research questions are:

What are the ICT policies in Australian gifted education programmes at the national and state levels?

Based on critical evaluations of ICT policies, how are these policies implemented across Australia?

The methodology used to answer these research questions is described in the section below.

## 4 Methodology

The guiding framework for this review is based on the work of Petticrew and Roberts [15] and Liberati et al. [16]. As such, this review implements a predefined review protocol, a comprehensive search process with strict inclusion criteria, and a critical appraisal of the evidence. The review considered a large number of records during the search process until thematic and data saturation indicated that no additional data collection was necessary to review [17]. The review sourced the Scopus and Web of Science and Google Scholar electronic databases on the 14th of April 2022, using the following search terms: (“Gifted education” OR “Gifted students” OR “ICT” OR “Policy” OR “ICT Policy” OR “Australia”) and: (“Gifted policy” OR “Australian Education”), and: (“Education” OR “l”) OR (“learning”).

### 4.1 Eligibility criteria

This review used narrow inclusion criteria, and every effort was made to ensure that the literature met minimum requirements. The inclusion criteria were defined as:

● **Content**: The search was limited to studies related to analyses of Australia's ICT policies in gifted education as well as gifted education policies in general. It contained the key words ‘Australia,’ ‘ICT policy,’ ‘gifted education,’ ‘gifted students,’ ‘gifted learners,’ ‘education,’ ‘technology,’ ‘analysis,’ ‘evaluation,’ and ‘review’ in the title or body of the article's abstract. The review was further limited to high-quality peer-reviewed journals (English only) as well as analysis reviews in the field of Education, particularly. When multiple articles from the same author appeared, these were excluded in an effort to eliminate bias.
● **Setting**: The scope of the review included those studies conducted only in traditional educational environments.
● **Participants**: The review is limited to studies evaluating policies related to gifted students in primary and secondary school. No further exclusions were implemented on the basis of ethnicity, gender, nationality, or other group identification.
● **Study Type**: The review considered only the literature for articles that evaluated the ICT policies for gifted education in Australia.
● **Exclusions**: After considering the above inclusion criteria, exclusions involved:
  1. Non-English publications
  2. Studies unrelated to Australia
  3. Studies unrelated to gifted education
  4. Studies unrelated to ICT policy
  5. Studies published before 2006
  6 Duplicate studies across databases

### 4.2 Search strategy and selection process

Despite the lack of a set criteria regarding how reviews should incorporate articles [16], the selection procedure should strive for objectivity and attempt to minimise errors [18]. The researcher independently identified studies for this review using the predefined eligibility protocol. The selection process included two iterations of screening, which resulted in an optimal balance of specificity and sensitivity.

This study utilised the most common databases among education scholars, such as Web of Science, SCOPUS, and Google Scholar, to source academic articles that review Australian ICT policy in gifted education. For the first iteration of screening, Boolean operators were used for combining and/or excluding specific terms, and for generating more effective search results. A list of search terms written in boolean script included: ‘Australia,’ ‘ICT policy,’ ‘gifted education,’ ‘gifted students,’ ‘gifted learners,’ ‘education,’ ‘technology,’ ‘analysis,’ ‘evaluation,’ and ‘review.’ This first phase yielded 205 records of article titles and abstracts.

Article titles and abstracts can provide readers with critical information about the scope of the study [16], which thus allowed the authors to determine eligibility for inclusion. The authors supposed that when the title and/or abstract included the keywords ‘ICT Policy’ or ‘Gifted education,’ the document was more likely to qualify for inclusion and need extra consideration in the second iteration.

**Fig. 1:**
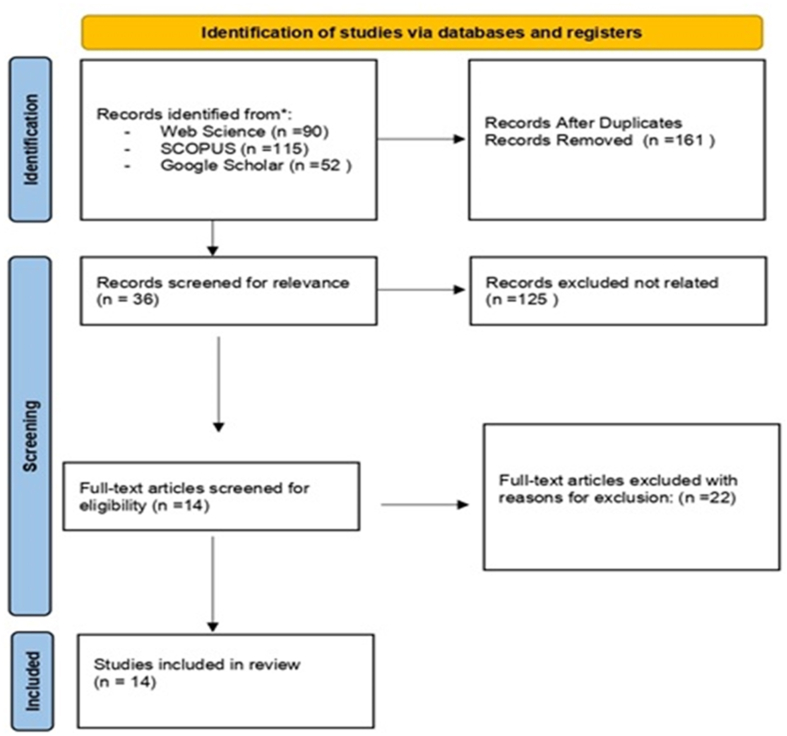
Prisma diagram.

The second screening iteration improved the screening process's sensitivity. The authors reviewed the thirty-six articles that had passed the first round of screening. The eligibility criteria were applied to the article titles, abstracts, and full text. There was no issue about how many documents were retained for review, and a list of the rejected articles and the reason for their exclusion was kept for record-keeping purposes. In summary, twenty-two articles were excluded in the second round of screening due to participant or study type disqualifications. This review considered fourteen articles in total (see Fig. 1).

## 5 Results & discussion

The results are divided into two sections, pertaining to each of the research questions. The first section discusses the current status of ICT policies of Australian national, state, and territorial governments, and the second section relates to critical evaluation of Australian ICT policies for gifted students.

### 5.1 Current status of ICT policies of Australian National, state and territorial governments

The current status of ICT policies in Australia can be understood by examining both national systemic policies and related policies involving ICT, such as Science, Technology, Engineering, and Mathematics (STEM), digital technologies, and distance education. The 2008 Melbourne Declaration on Educational Goals for Young Australians, which outlines the Australian educational curriculum, emphasises equity and excellence and addresses the educational needs of gifted students [19]. The description of learning areas and general capabilities further expand on these goals, including considerations for cross-cultural implications and admission policies for gifted Aboriginal and Torres Strait Islander students. While these policies recognise the importance of meeting the needs of gifted students, the explicit mention of ICT in the school environment is lacking.

STEM policies, falling under the ICT umbrella, are also relevant to the discussion of ICT policies. Timms et al. [20] critically reviewed Australian STEM education strategies for 2016–2023, which include elements related to ICT policies. These strategies aim to enhance 10.13039/100010424STEM competence among students, develop teacher capacity to deliver 10.13039/100010424STEM education, and provide support to schools. Gifted students, who frequently continue their educational journey in STEM fields, are inherently encompassed within STEM policies, which also involve policies for gifted education and the integration of ICT elements. The report provides a comprehensive list of STEM policies targeting students, teachers, and curriculum, with a significant number of them directly related to ICT and accompanied by relevant resource links. Notably, the policy on summer schools for students incorporates ICT initiatives called DigIT and Curious Minds. In terms of instructional support, there are programs like CSER Digital Technologies Education programmes and digital literacy school grants aimed at training teachers in essential ICT skills. In addition, the curriculum encompasses a digital technologies curriculum, computer coding, and Australian Digital Technology challenges for Year 5 and 7 students.

In rural and remote locations, distance education has been utilised since the early 1900s, with ICT increasingly facilitating synchronous and asynchronous learning interactions [21]. Downes, Roberts, and Barbour [22] observed the inclusion of gifted students in these innovative practices in New South Wales (NSW), supported by state and local administration policies. Duff [23] investigated provisions and support measures for gifted and talented students in rural and remote areas of Queensland, which also included ICT policies.

However, policy deficits have been identified in addressing the needs of teachers in rural and remote areas, particularly in utilising ICT to teach gifted and talented students and Indigenous students [24]. The limited facilities in these areas can often necessitate combining classes, which is more prevalent in rural and remote locations than in metropolitan areas.

The Northern Territory policy document on education of gifted and talented students [25] includes the use of digital technologies in teaching. The Australian Curriculum [26] describes the curricular requirements for gifted and talented students and provides state-wise information, but does not explicitly highlight the need to use ICT in teaching these students.

These findings highlight the varied landscape of ICT policies in Australia, emphasising the importance of addressing the specific needs of gifted students within national, STEM, distance education, and rural/remote education policies. The integration of ICT into these policies is crucial to support the educational experiences of gifted students and ensure their inclusion and access to appropriate learning opportunities.

### 5.2 Critical evaluation of the Australian ICT policies for gifted students

This section provides critical evaluation of the Australian ICT policies for gifted based on various studies and reports. The initial review conducted by Batanero, Rebollo, and Rueda [27] focused on the impact of ICT on gifted students rather than the policies themselves. While this review served as a starting point, it revealed a scarcity of studies on the use of ICT to support gifted students. However, it acknowledged the scientific impact of ICT in Australia, Europe and North America. Most papers reviewed utilised qualitative methods with reflective analysis.

One crucial aspect of the Australian ICT policies for gifted students is the disparity faced by Aboriginal gifted students in rural areas regarding access to ICT. Townend, Hay, Jung, and Smith [28] emphasised the disadvantaged position of these students despite specific policy initiatives by local, state, and national governments. Similarly, Sazali, Franklin, Dillon, and Yeung [29] highlighted the need for prioritised policies that address the required ICT infrastructure and technical support for Aboriginal gifted students.

The TIMSS results of 2019 shed light on Australia's ranking in STEM subjects. While there was improvement in Year 8 mathematics and science as well as Year 4 science, Year 4 mathematics showed no progress since 2007. According to a 2020 report by the Australian Council for Educational Research (ACER), only 68–78% of Australian students achieved the international intermediate STEM proficiency standard, in contrast to leading countries like Singapore where more than 90% of students reached this standard. ICT has been identified as an essential component in improving students' performance in STEM subjects nationally and internationally, as discussed by Timms, Moyle, Weldon, and Mitchell [30]. Despite the implementation of ICT policies, there is still a long way to go in achieving the desired level of proficiency in STEM.

Examining the existing ICT policies mentioned in the 2020 ACER report, it becomes apparent that only a few states and territories (e.g., Australian Capital Territory, Queensland, Victoria, and Western Australia) have specified policies for students, teachers, and curriculum. The declining performance in ICT literacy since 2011 and the varying policies for assessing ICT literacy pose barriers to developing an integrated ICT policy. There is a need to emphasise ICT literacy in STEM curricula and bridge the gap between traditional and STEM subjects. Efforts have been made to integrate technology into the curriculum and address real-world problems through interdisciplinary STEM curricula. Nevertheless, effectively implementing these policies is not a straightforward task, demanding targeted teacher training and careful planning to ensure consistent outcomes.

In considering the use of ICT for teaching and learning among gifted students, Bannister, Cornish, Bannister-Tyrrell, and Gregory [31] highlighted the virtual high school environments in New South Wales (NSW), where gifted students focus on three core subjects through virtual high school environments while learning other subjects in their local schools. This approach demonstrates the influence of policies on utilising ICT for the education of gifted students.

While ICT plays a significant role in enhancing education for gifted students, it is important to strike a balance. The School of Education at the University of New England, with funding SiMEER NSW, developed an innovative enrichment program for gifted rural and regional school students. This program incorporated ICT to assess problem-solving abilities, animation design skills, and knowledge of specific subjects among gifted students [32]. On the other side of this policy [33], cautioned against over-reliance on ICT and advocated for the creation of personalised educational environments that encourage interactions with peers, teachers, and mentors. This perspective is reflected to a great extent in the Australian ICT policies for gifted education by not emphasising ICT too heavily in the policy statements (see the statements in ACT, 2021).

Furthermore, Australia recognises the importance of ICT in work and daily life, as highlighted in the ACER report. As part of the National Assessment Program, ICT literacy has been integrated into the assessment program conducted by ACER on behalf of the national government. The report revealed that Year 6 students have shown notable progress since the last assessment in 2005, while Year 10 students demonstrated only slight improvement. To facilitate ICT learning in challenging rural conditions, Australia launched the One Laptop per Child program, which has yielded positive results. Additionally, ACER established the Digital Education Research Network (DERN) to foster research on the use of digital technologies in education. Although these initiatives were not specifically targeted at gifted students, many of the ICT strategies driven by these policies have benefitted gifted students as well [34].

A comprehensive review of policies across different states and territories of Australia regarding the education of gifted students conducted by Walsh and Jolly [35] identified both similarities and differences. Each state and territory education department has its own unique policies, all of which define giftedness based on Gagné’s Differentiated Model of Giftedness and Talent. However, despite some similarity in the use of certain IQ tests and other procedures, there is generally a lack of uniformity in identifying gifted students among states, school sectors, and individual schools. While a list of policies for gifted children exists for six states and territories, none of the policy statements mention an ICT policy for education of gifted children. However, notable successes in virtual schooling for gifted students have been observed in NSW. Among state programs, Tasmania stands out for its government-run online extension enrichment program, which attends to the education of gifted students from preparatory to Year 8 stages. The Tasmanian Department of Education also offers gifted online courses in various subjects [36].

The training and development of teachers in utilising ICT for teaching gifted students is another essential aspect of ICT policies. Hardy [37] noted that Australian Federal policies implemented during 1996–2007 by the ruling government at the time have significantly supported this requirement. Similarly, the report by Timms, Moyle, Weldon, and Mitchell [20] highlights the need for teaching training policies in the context of STEM education for school students in Australia. Interviews with 22 pre-service teachers undergoing training at a regional campus of a Queensland university conducted by Watters, Hudson, and Hudson [38] identified ICT as one of the teaching strategies recognized by these future educators for meeting the needs of gifted students.

In summary, the evaluation of Australian ICT policies for gifted students reveals both strengths and areas for improvement. While the integration of ICT literacy into the National Assessment Program and the implementation of initiatives like the One Laptop per Child program demonstrate the recognition of ICT's importance, there is still a lack of explicit ICT policies for gifted education. The diversity in policies across states and territories underscores the need for greater consistency in identifying and catering to the needs of gifted students. In addition, the training and development of teachers to effectively utilise ICT in teaching gifted students is crucial for ensuring their educational success.

## 6 Conclusions

Although educational policies for gifted students can be found in national, state, and territorial government documents, no single standard for identifying gifted students or providing gifted education is equally applied across Australia. Furthermore, few explicit statements or policies about ICT in gifted education have been specified by state governments. Some policies only imply use of ICT to benefit learning for gifted students in rural and remote locations, of whom the majority includes Aboriginal and Torres Strait Islanders. Absence of clearly spelt-out ICT policies in government documents is a major limitation to ensuring equal access to high quality gifted education for all people in Australia.

Based on the discussion of ICT policies for gifted students in Australia and the identified gaps and limitations, here are some recommendations:

1. Implement consistent identification processes: Establish clear and consistent procedures for identifying gifted students, ensuring that identification practices are fair, transparent, and inclusive. These processes should incorporate multiple measures to identify students' strengths and talents across various domains, including intellectual, creative, artistic, and leadership abilities.
2. Explicitly integrate ICT in gifted education policies: Revise national systemic policies and frameworks, such as the 2008 Melbourne Declaration on Educational Goals for Young Australians, to explicitly acknowledge the importance of ICT in supporting the educational needs of gifted students. This can ensure that ICT is recognized as an essential tool for personalised learning, enrichment, and engagement.
3. Develop targeted professional development programs: Implement comprehensive professional development programs for teachers that specifically focus on utilising ICT to meet the needs of gifted students. These programs should provide training on incorporating advanced technologies, digital resources, and online collaboration platforms to create challenging and stimulating learning environments for gifted students.
4. Expand access to ICT resources in rural and remote areas: Address the existing policy deficits in rural and remote areas by allocating resources and infrastructure to improve ICT access. This includes providing reliable internet connectivity, adequate hardware and software, and technical support to schools in these areas. Efforts should be made to bridge the digital divide and ensure equitable opportunities for gifted students in all geographical locations.
5. Foster partnerships and collaborations: Encourage collaboration between educational institutions, government agencies, and industry partners to support the integration of ICT in gifted education. Foster partnerships that provide access to cutting-edge technologies, mentorship programs, and real-world experiences for gifted students. These collaborations can enhance the learning opportunities and expose gifted students to emerging fields and career pathways.
6. Develop ICT guidelines for gifted education: Create specific guidelines or frameworks that outline best practices for incorporating ICT in gifted education. These guidelines can offer recommendations on selecting appropriate digital resources, designing challenging projects, and leveraging online platforms for collaboration and knowledge sharing among gifted students.
7. Support research and innovation: Allocate resources to support research and innovation in the field of ICT-enabled gifted education. Encourage studies -- particularly longitudinal in nature -- that explore effective strategies, evaluate the impact of ICT integration, and identify emerging technologies and trends that can enhance educational experiences for gifted students. Additional qualitative data is necessary to shed light on the emic perspectives of various stakeholders, such as gifted students and their parents, to understand how to better serve the recipients of gifted education themselves.

By implementing these recommendations, policymakers and educators can better address the specific needs of gifted students within ICT policies, promote their inclusion, and provide them with enhanced learning opportunities that foster their full potential.

## Ethics consecrations

An ethics statement is not applicable because this study is based exclusively on published literature.

## Production notes

### Author contribution statement

All authors listed have significantly contributed to the development and the writing of this article.

### Data availability statement

No data was used for the research described in the article.

## Declaration of competing interest

The authors declare that they have no known competing financial interests or personal relationships that could have appeared to influence the work reported in this paper.ss

## References

[1] Davis G.A., Sylvia B. (2011).

[2] ICEF (2019, November 18). https://monitor.icef.com/2019/11/australian-international-student-enrolments-up-11-through-september-2019/.

[3] Gagné F. (1985). Giftedness and talent: reexamining a reexamination of the definitions. Gift. Child. Q..

[4] Braggett E.J., Day A.H., Minchin M. (1997).

[5] Feldhusen J.F., Proctor T.B., Black K.N. (1986 Sep 1). Guidelines for grade advancement of precocious children. Roeper Rev..

[6] Eren F., Çete A.Ö., Avcil S., Baykara B. (2018). Emotional and behavioral characteristics of gifted children and their families. Arch. Neuropsych..

[7] NAGC (2021). https://www.nagc.org/resources-publications/resources/what-giftedness.

[8] Soloway E., Pryor A. (1996 Apr 1). The next generation in human-computer interaction. Commun. ACM.

[9] Tinio V. (2003). https://www.apdipnet/publications/lespprinmers/eprimenredu.edu.pdf.

[10] Riley T., Sturgess A. (2005). Professional development to support gifted and talented education in New Zealand. Aus. J. Gif. Edu..

[11] Asher W. (2003). Meta-analysis and gifted education. J. Educ. Gift..

[12] Sheffield C.C. (2007). Technology and the gifted adolescent: Higher order thinking, 21st century literacy, and the digital native. Meridian: Midd. Sch. Comp. Tech. J..

[13] Stratford R., Brown M. (2002). Towards a political understanding of New Zealand's ICT strategies. Comp. New Zealand Sch..

[14] Kozma R. (2011).

[15] Petticrew M., Roberts H. (2008 Apr). Systematic reviews–do they ‘work’in informing decision-making around health inequalities?. Health Econ. Pol. Law.

[16] Liberati A., Altman D.G., Tetzlaff J., Mulrow C., Gøtzsche P.C., Ioannidis J.P., Clarke M., Devereaux P.J., Kleijnen J., Moher D. (2009). The PRISMA statement for reporting systematic reviews and meta-analyses of studies that evaluate health care interventions: explanation and elaboration. Ann. Intern. Med..

[17] Saunders B., Sim J., Kingstone T., Baker S., Waterfield J., Bartlam B., Burroughs H., Jinks C. (2018 Jul). Saturation in qualitative research: exploring its conceptualization and operationalization. Qual. Quantity.

[18] Higgins J.P., Thomas J., Chandler J., Cumpston M., Li T., Page M.J., Welch V.A. (2019 Sep 23). Cochrane Handbook for Systematic Reviews of Interventions.

[19] Farrall J., Henderson L. (2015).

[20] Timms M.J., Moyle K., Weldon P.R., Mitchell P. (2018).

[21] Wong G.K., Yang M. (2017).

[22] Downes N., Roberts P., Barbour M. (2020).

[23] Duff J. (2020 Dec). Provisions for gifted and talented students in Queensland rural and remote high schools. Aus. J. Gif. Edu..

[24] Lyons T., Cooksey R., Panizzon D., Parnell A., Pegg J. (2006 Jul).

[25] NTBoS (2018). https://education.nt.gov.au/__data/assets/pdf_file/0005/513419/T-12-CurriculumPedagogyAssessmentReportingFramework.pdf.

[26] Australian Curriculum (2021). https://www.australiancurriculum.edu.au/resources/student-diversity/meeting-the-needs-of-gifted-and-talented-students/.

[27] Batanero J.M.F., Rebollo M.M.R., Rueda M.M. (2019). Impact of ICT on students with high abilities. Bibliographic review (2008–2018). Comput. Educ..

[28] Townend G., Hay P.K., Jung J.Y., Smith S.R. (2021). Handbook of Giftedness and Talent Development in the Asia-Pacific.

[29] Sazali S., Franklin A., Dillon A., Yeung A.S. (2020).

[30] Timms M.J., Moyle K., Weldon P.R., Mitchell P. (2018).

[31] Bannister B., Cornish L., Bannister-Tyrrell M., Gregory S. (2015). Creative use of digital technologies: keeping the best and brightest in the bush. Aus. Int. J. Rur. Edu..

[32] Smith S., Smith R. (2009). Enhancing rural and regional gifted student experiences: exemplars of innovative enrichment practice. Improv. Equ. Rur. Edu..

[33] Smith S.R., Laura R.S. (2009). Repersonalizing educational ecologies to nurture the social and affective needs of gifted children. Asia-Pacific J. Gifted Tal. Edu..

[34] Masters G. (2010). https://research.acer.edu.au/cgi/viewcontent.cgi?article=1077&context=resdev.

[35] Walsh R.L., Jolly J.L. (2018). Gifted education in the Australian context. Gift. Child Today.

[36] Department of Education T. (2021). https://www.education.tas.gov.au/parents-carers/parent-fact-sheets/gifted-students/.

[37] Hardy I. (2008). Competing priorities in professional development: an Australian study of teacher professional development policy and practice. Asia Pac. J. Teach. Educ..

[38] Watters J.J., Hudson S., Hudson P. (2013). Orienting preservice teachers towards gifted education: schooluniversity partnerships. Aus. J. Gif. Edu..

